# Chronic Effects of Maternal Low-Protein and Low-Quality Protein Diets on Body Composition, Glucose-Homeostasis and Metabolic Factors, Followed by Reversible Changes upon Rehabilitation in Adult Rat Offspring

**DOI:** 10.3390/nu13114129

**Published:** 2021-11-18

**Authors:** Pandarinath Savitikadi, Raghu Pullakhandam, Bharati Kulkarni, Boiroju Naveen Kumar, Geereddy Bhanuprakash Reddy, Vadde Sudhakar Reddy

**Affiliations:** 1 Biochemistry Division, ICMR-National Institute of Nutrition, Hyderabad 500007, India; pandarinath12@gmail.com (P.S.); raghu_nin2000@yahoo.com (R.P.); geereddy@yahoo.com (G.B.R.); 2 Clinical Division, ICMR-National Institute of Nutrition, Hyderabad 500007, India; dr.bharatikulkarni@gmail.com; 3 Biostatistics Division, ICMR-National Institute of Nutrition, Hyderabad 500007, India; nanibyrozu@gmail.com

**Keywords:** low-quality protein, low-protein, wheat gluten, growth, rehabilitation, body composition, glucose homeostasis

## Abstract

Several studies suggest that the maternal protein content and source can affect the offspring’s health. However, the chronic impact of maternal quality and quantity protein restriction, and reversible changes upon rehabilitation, if any, in the offspring, remains elusive. This study examined the effects of maternal low-quality protein (LQP) and low-protein (LP) intake from preconception to post-weaning, followed by rehabilitation from weaning, on body composition, glucose-homeostasis, and metabolic factors in rat offspring. Wistar rats were exposed to normal protein (NP; 20% casein), LQP (20% wheat gluten) or LP (8% casein) isocaloric diets for 7 weeks before pregnancy until lactation. After weaning, the offspring were exposed to five diets: NP, LQP, LQPR (LQP rehabilitated with NP), LP, and LPR (LP rehabilitated with NP) for 16 weeks. Body composition, glucose-homeostasis, lipids, and plasma hormones were investigated. The LQP and LP offspring had lower bodyweight, fat and lean mass, insulin and HOMA-IR than the NP. The LQP offspring had higher cholesterol, T3 and T4, and lower triacylglycerides and glucose, while these were unaltered in LP compared to NP. The majority of the above outcomes were reversed upon rehabilitation. These results suggest that the chronic exposure of rats to maternal LQP and LP diets induced differential adverse effects by influencing body composition and metabolism, which were reversed upon rehabilitation.

## 1. Introduction

Extensive epidemiological reports and studies in animal models have shown that a sub-optimal environment during fetal and early life development influences the adulthood risk of metabolic diseases, including obesity and type-2 diabetes [1,2,3,4]. Several dietary manipulations including caloric restriction [5] and the restriction of specific macronutrients during pregnancy [6] had permanent effects on offspring health. Protein–energy malnutrition during pregnancy and childhood remains a major contributor to impaired growth and development in developing countries [7]. In line with epidemiological observations in humans, experimental studies in rodents show that a maternal low-protein (MLP) diet or dietary protein dilution is associated with numerous diseases, including obesity [8], diabetes [9,10], hypertension [11], and cardiovascular disease [12] in the offspring. Furthermore, the MLP diet causes altered mammary gland development and function [13] and milk composition [14]. Maternal protein restriction (MPR) during gestation and lactation changed the body composition by decreasing bodyweights (BWs), visceral fat mass, and total body fat in rat offspring [15]. Along with the protein content in the diet, the protein quality in the maternal diet influenced the BWs and was speculated to impact the risk of developing metabolic diseases in the offspring [16,17]. Animal and plant-derived diets differ in their indispensable amino acid (IAA) content and digestibility [18]. Several past studies indicated significant differences in the intake of protein and IAA between developed and developing countries [19,20,21,22]. India’s typical diets are predominantly cereal/pulse-based, and in fact, cereals contribute 60% of the dietary protein intake in rural India [23].

The quality of cereal/legume protein is lower than that of animal-source foods [24,25], and the intake of low-quality protein (LQP) is linked with stunting in Indian children [25]. An analysis of diet surveys in India indicated that the risk of LQP intake varies between 4% and 26% in different age groups and urban and rural Indians [26]. Poor socioeconomic environments or increased demand due to persistent intestinal infections exacerbate the risk of LQP from 6% to 42%. These studies together suggest a substantial risk of protein inadequacy in the Indian population. The effects of different dietary proteins, including animal and plant proteins, on blood pressure (BP) were studied in a normal Dutch population, and it was found that animal proteins were not associated with BP, while plant proteins were inversely associated with BP [27]. The consumption of different quality proteins—including casein, soy, and whey—for seven consecutive days led to a decrease in satiety, an increase in diet-induced thermogenesis, and a decrease in the respiratory quotient in a normal-weight Brazil population [28]. Furthermore, the ingestion of different protein meals—including whey, tuna, turkey, and egg albumin—in randomized lean and healthy men induced a greater insulin response with whey compared to other proteins [29].

Few studies have investigated whether LQP diets such as wheat gluten (WG) as the protein source reduced the biomechanical properties of femoral bone and mandibles in adult rats [30,31]. Maternal LQP diet has been shown to alter the plasma amino acid levels [32] and expression of hepatic methyltransferases in rat offspring [33]. Offspring born to mothers fed with soy protein during gestation and lactation have a higher food intake and BWs compared to offspring born to mothers fed with casein protein [17]. Maternal diets of different protein quality—including peanut and turkey protein—during pregnancy and lactation reduced the bodyweight compared to milk protein in the offspring [34]. Experimental MPR studies focused on either gestation or lactation alone, or combined periods of gestation and lactation, do not emulate the human condition, where undernutrition is not confined to fetal life but, rather, is a chronic or life-long condition. The chronic protein restriction (PR) from the combined prenatal to the postnatal stage may have different programming effects on the body phenotype than the PR during gestation and/or lactation only. Furthermore, little is known about whether the impact of the MLP diet mimics or differs from that of the MLQP diet regarding outcomes.

Therefore, apart from understanding the effect of MLP and MLQP intake on offspring metabolic status, it is important to study whether these changes are reversible after the restoration of optimal diets, as this will have implications in both complementary feeding and maternal and infant supplementary nutrition programs in developing countries. Therefore, in the present study, we evaluated the chronic effect of MLQP and MLP diets from combined periods of preconception to post-weaning with respect to body composition, glucose homeostasis, and metabolic factors, and examined the impact of rehabilitation on the aforementioned changes.

## 2. Materials and Methods

### 2.1. Animals and Study Design

All of the procedures involving animal experiments were approved by the Institutional Animal Ethical Committee (ref # ICMR-NIN/IAEC/01/001/2019) of the ICMR-National Institute of Nutrition (ICMR-NIN). Female Wistar-NIN (WNIN) rats of approximately 90 days of age were obtained from the animal facility of ICMR-NIN, Hyderabad, India. The animals were housed in individual cages, and were maintained at the ambient temperature (22 ± 2 °C, 50% humidity, and a 12 h light/dark cycle with lights on at 08:00). The animals were maintained on a standard laboratory chow diet, and were acclimatized to the laboratory conditions one week before the start of the dietary regimens.

#### 2.1.1. Prepregnancy and Gestation

After one week of acclimatization, the animals were randomly assigned to be fed an AIN93-based isocaloric diet containing either NP (20% casein, *n* = 4) or LQP (20% WG, *n* = 4), or a restricted protein or low-protein diet (LP; 8% casein, *n* = 4) for 7 weeks. We chose 8% casein as the LP diet, as it was considered to be a moderate protein restriction [35]. After 7 weeks, the female rats were mated with male rats in a proportion of 2:1, and the presence of a mating plug was checked early in the morning. The appearance of the mating plug was designated as day 0 of pregnancy. The dams were housed in individual cages, were provided with food and water ad libitum, and continued on their respective diets throughout the experiment. All of the diets were formulated as per AIN-93G guidelines which were specifically designed for growth, pregnancy, and lactation [36].

#### 2.1.2. Birth and Lactation

At birth, the litter size, sex, and pup weight were recorded, and the day of delivery was designated as day 0 of postnatal life. In order to ensure homogeneity, the litters were normalized to nine pups wherever possible.

#### 2.1.3. Weaning and Post-Weaning

**Figure 1:**
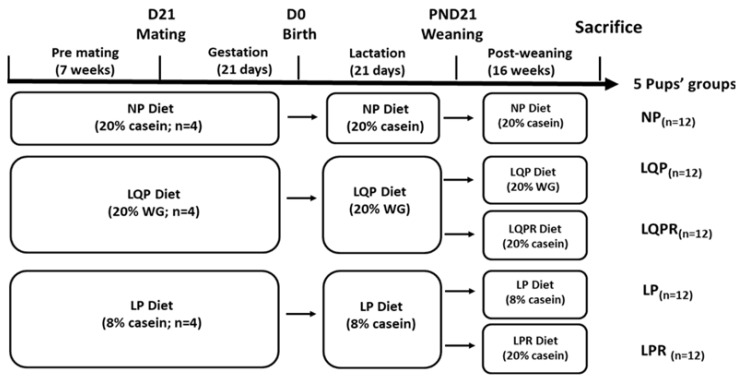
Experimental design: Female WNIN rats were alimented with either a normal protein (NP) diet with 20% casein (*n* = 4) or a low-quality protein (LQP) with wheat gluten (WG) diet (*n* = 4), or a low-protein (LP) diet with 8% casein (*n* = 4) for 7 weeks, followed by mating with males. All of the animals received the same diet throughout gestation and lactation. At the weaning, the offspring were assigned into the following groups: NP diet (*n* = 12), LQP diet (*n* = 12), LQPR diet (a group of offspring from the LQP group rehabilitated with an NP diet; *n* = 12), LP diet (*n* = 12), LPR diet (a group of offspring from the LP group rehabilitated with the NP diet; *n* = 12). The offspring were sacrificed after maintaining their respective diets for 16 weeks after weaning.

At weaning (postnatal day 21), both the male and female offspring weight was recorded. After weaning, the offspring were divided into the following groups: offspring born to mothers fed with an isocaloric diet containing either NP (*n* = 12) or LQP (*n* = 12), or a group of animals from the LQP group rehabilitated with the NP diet for recovery (LQPR; *n* = 12) or the LP diet (LP; *n* = 12), or a group of animals from the LP group rehabilitated with the NP diet for recovery (LPR; *n* = 12), and were maintained on their respective diets for 16 weeks. The preliminary analysis revealed no sex-dependent statistical significance of weight at birth or weaning. Therefore, we selected both males and females from each litter. The food intake of the mothers and offspring were recorded daily. The offspring were housed individually, and food was provided in the form of a powder diet in the cups. A weighed excess of food was provided each day, and the amount remaining after 24 h was weighed. The whole animal experiment design is depicted in Figure 1. The diets were isocaloric on metabolizable energy, and their composition is described in Table 1.

### 2.2. Whole-Body Composition Assessment by Dual-Energy X-ray Absorptiometry (DEXA)

The animal’s whole-body composition and bone health were assessed using DEXA scanning (QDR Discovery A; S/N 82382, Hologic, Waltham, MA, USA). The animals were briefly immobilized with xylazine and isoflurane anaesthesia during the 2–3 min duration of the scan to determine the parameters, including the total body surface area, bone mineral content (BMC), bone mineral density (BMD), total mass, lean mass, fat mass, percent lean body mass (% LBM), and percent body fat (% BF). The scans were analyzed using the rat whole-body software (v.13.3.0.1). DEXA was conducted twice after 4 weeks from the start of the experimental diet, and at weaning in the mothers. Meanwhile, DEXA was conducted at weaning, and 10 weeks after weaning in the offspring.

### 2.3. Body and Organ Weights

The maternal and offspring BWs were recorded weekly from the start of the experiment. The mothers were sacrificed one month after weaning, while the offspring were sacrificed after maintaining their respective diets for 16 weeks. The animals fasted overnight, and a fasting blood sample was collected via retro-orbital plexus between 08.00 and 09.00 h for the plasma analysis. The body organs were collected, weighed, snap-frozen in liquid nitrogen, and stored at −80 °C for further analysis.

### 2.4. Biochemical Analysis

The blood samples were collected in EDTA capillary tubes and centrifuged at 3000 rpm for 20 min, at 4 °C, to obtain the plasma. The plasma was collected and stored at −80 °C for further analysis. The fasting glucose (FG) levels were measured by the glucose oxidase–peroxidase (GOD–POD) method using commercially available kits (#11504; Biosystems, Barcelona, Spain) in the plasma. The insulin levels were estimated using the commercially available sandwich ELISA kit (#10-1250-01, Mercodia, Uppsala, Sweden). The homeostatic model assessment of the insulin resistance (HOMA-IR) was determined using the following formula: insulin (μIU/mL) x serum glucose (mmol/l)/22·5. The lipid profile, including triacylglycerides (TAG; #11529) and cholesterol (#11505), was estimated using commercially available kits (Biosystems, Barcelona, Spain). The alkaline phosphatase (ALP) levels were assessed using commercially available kits (#11593; Biosystems, Barcelona, Spain). Hemoglobin (Hb) was estimated using Drabkin’s method. The plasma T3 (#ITER00534) and T4 (#IT7324) levels were assessed using the commercially available competitive ELISA kit (ImmunotagTM, coated with antigen, G-Biosciences, St Louis, MO, USA). The cortisol (# ITER00828) and leptin (#ITER00561) levels were estimated using the commercially available sandwich ELISA kit (ImmunotagTM, G-Biosciences, St Louis, MO, USA).

### 2.5. Statistical Analysis

The effect of the maternal diet on the offspring was assessed using the one-way analysis of variance (ANOVA), and multiple comparisons were assessed using the Tukey post hoc test. Two-way ANOVA was performed to determine the food and energy intake, BWs, and the interaction between the maternal diet and sex. The statistical analyses were performed using SPSS software 19. The differences between the groups were considered significant at *p* < 0.05. All of the data are expressed as means ± S.D.

## 3. Results

### 3.1. Effects of the LQP and LP Diets on the Dams

#### 3.1.1. Food Intake and BWs in the Mothers

**Figure 2:**
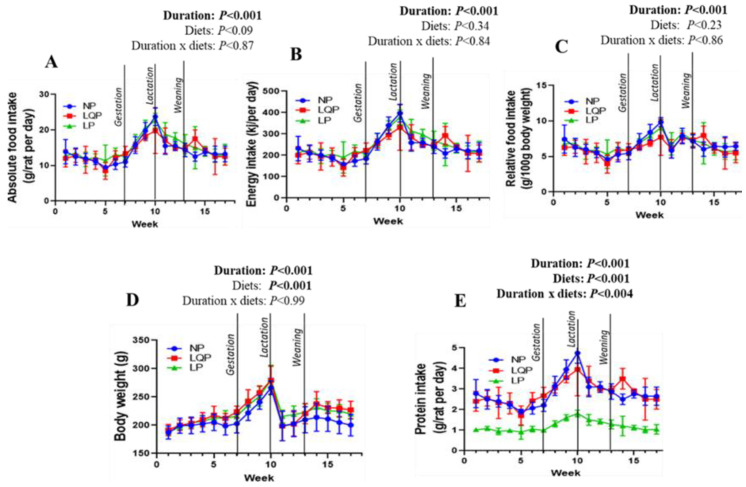
Effect of protein restriction on food intake and bodyweights (BWs) in mothers: the food intake was recorded daily, while BWs were recorded weekly. (**A**) Absolute food intake, (**B**) energy intake, (**C**) relative food intake, and (**D**) BWs (**E**) Protein intake. The data were tested using two-way ANOVA, and are represented as the mean ± SD (*n* = 4 per group). NP, normal protein; LQP, low-quality protein; LP, low-protein.

The absolute food intake was significantly affected by study duration (*p* < 0.001) but not the type of diet (Figure 2A). The energy intake and relative food intake were also significantly affected only by the study duration (*p* < 0.0001; Figure 2B,C). Bodyweight was significantly affected by the study duration (*p* < 0.001) and diet (*p* < 0.001; Figure 2D). The bodyweight of the LQP (*p* < 0.0001) and LP mothers (*p* < 0.0003) was significantly higher than that of the NP mothers (Figure 2D). The average protein intake was significantly influenced by the study duration (*p* < 0.001) and diet (*p* < 0.001) (Figure 2E).

#### 3.1.2. Body Composition and Metabolic Profile of the Mothers

The body composition parameters—including the total body surface area, BMD, BMC, total mass, lean mass, % LBM, fat mass, and % BF—were comparable between the groups at 4 weeks (Table 2). Nonetheless, the % LBM, fat mass, and % BF were significantly altered at weaning. The percent LBM (because of higher bodyweight) was significantly lower in LQP (*p* < 0.004) and LP (*p* < 0.001), while the fat mass was higher in LQP (*p* < 0.05) and LP (*p* < 0.004) than the NP mothers. In the same line, % BF was significantly higher in LQP (*p* < 0.004) and LP (*p* < 0.001) than the NP mothers. The FG levels were comparable between the NP, LQP, and LP mothers (Table 2).

#### 3.1.3. Mating Success

All of the animals in the NP, LQP, and LP groups became pregnant and delivered the pups, except one animal in the LQP group, while one animal in the LP group delivered only one pup. There was no significant difference in litter size between the NP (8.75 ± 0.95, *n* = 4), LQP (10.33 ± 3.78, *n* = 3), and LP (10 ± 1; *n* = 3) groups. In total, 96 pups were born: the NP dams delivered 35 pups (males: *n* = 10, females *n* = 25), the LQP dams delivered 31 pups (males: *n* = 17, females: *n* = 14), and the LP dams delivered 30 pups (males: *n* = 16, females: *n* = 14).

### 3.2. Effect of MLQP and MLP Diets on the Offspring

#### 3.2.1. Bodyweight of the Pups at Birth and Weaning

**Figure 3:**
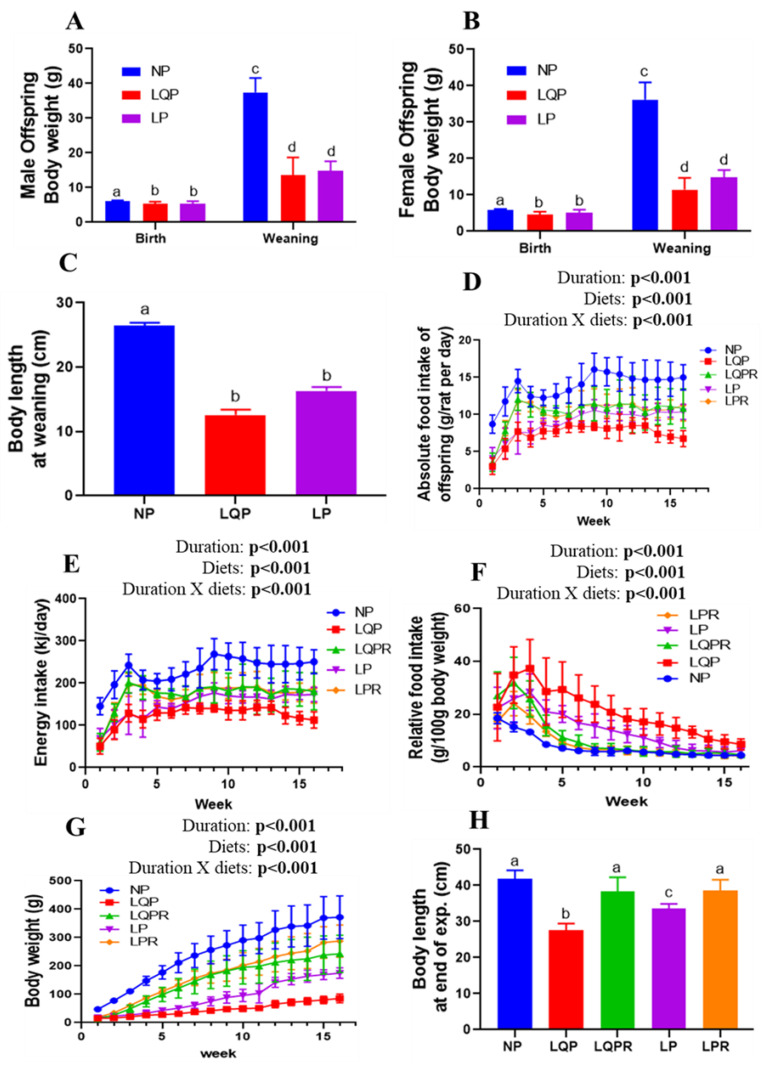
Effect of maternal protein restriction followed by rehabilitation on bodyweight, food intake, and body length in the offspring: (**A**) bodyweight of the male pups at birth (NP, *n* = 10; LQP, *n* = 17; LP, *n* = 16) and weaning (NP, *n* = 10; LQP, *n* = 14; LP, *n* = 16); (**B**) BWs of the female pups at birth (NP, *n* = 22; LQP, *n* = 14; LP, *n* = 13) and weaning (NP, *n* = 20; LQP, *n* = 10; LP, *n* = 10); (**C**) body length of the pups at weaning (NP, *n* = 12; LQP, *n* = 8–12, LP, *n* = 11–12); (**D**) absolute food intake (NP, *n* = 12; LQP, *n* = 8–12, LQPR, *n* = 11–12, LP, *n* = 11–12; LPR, *n* = 12); (**E**) energy intake; (**F**) relative food intake; (**G**) weekly BWs of the offspring from weaning to the end of the experiment (NP, *n* = 12; LQP, *n* = 8–12, LQPR, *n* = 11–12, LP, *n* = 11–12; LPR, *n* = 12); (**H**) body length of the offspring at the end of the experiment (*n* = 6 per group). The data were tested using two-way ANOVA and one-way ANOVA. The bars indicate the mean ± SD, and the bars that share different superscript letters differ significantly (*p* < 0.05). NP, normal protein; LQP, low-quality protein diet; LQPR; low-quality protein group rehabilitated with the NP diet; LP, low-protein; LPR, low-protein group rehabilitated with the NP diet.

The birth weight of the pups was significantly affected by diet (*p* < 0.0001) and sex (*p* = 0.0007; Figure 3A,B). However, there was no significant interaction of diet and sex with birth weight (*p* = 0.29). The bodyweight of the male pups was significantly lower in LQP (13%; *p* < 0.01 and 63%; *p* < 0.001), and LP (13%; *p* < 0.01 and 63%; *p* < 0.001), at birth and weaning than the NP offspring respectively (Figure 3A). Similarly, the BWs of the female pups were significantly lower in LQP (20%; *p* < 0.001 and 68%; *p* < 0.001), and LP (11%; *p* < 0.01 and 59%; *p* < 0.001) at birth and weaning than the NP offspring, respectively (Figure 3B). At weaning, the BWs of the offspring were significantly affected by diet (*p* < 0.0001) but not sex (*p* = 0.21). Furthermore, there was no significant interaction of diet and sex with BW at weaning (*p* = 0.61). The body length of the offspring at weaning was significantly lower in LQP (*p* < 0.0001) and LP (*p* < 0.0001) than NP offspring (Figure 3C).

#### 3.2.2. Food Intake and BWs of the Offspring from Weaning to End of the Experiment

The absolute food intake of the offspring from weaning to the end of the experiment was significantly affected by the study duration (*p* < 0.001) and diet (*p* < 0.001; Figure 3D). The absolute food intake of LQP (*p* < 0.0001) and LP (*p* < 0.0001) was significantly lower than that of the NP offspring (Figure 3D). However, the food intake of the LQPR offspring significantly increased from the second week compared to LQP (*p* < 0.0001), while the LPR offspring also showed a significant increase in food intake compared to LP (*p* < 0.0001; Figure 3D). Energy intake was also substantiated with absolute food intake, and was significantly affected by diet (*p* < 0.0001) and the study duration (*p* < 0.0001; Figure 3E). The relative food intake was significantly greater in LQP followed by LP than in the NP offspring across the first few weeks. However, this difference decreased across subsequent weeks, and was comparable between the groups at the end of the experiment (Figure 3F). The relative food intake was significantly affected by diet (*p* < 0.0001) and study duration (*p* < 0.0001). After weaning, the BWs of LQP grew very slowly, followed by LP, compared to the NP offspring (Figure 3G). The bodyweight of the offspring was significantly influenced by the study duration (*p* < 0.001) and diet (*p* < 0.001; Figure 3G). At the end of the experiment, the BWs were drastically lower in LQP (75%; *p* < 0.0001) and LP (52%; *p* < 0.0001) than the NP offspring. However, the BWs were significantly higher in LQPR (44%; *p* < 0.0001) and LPR (29%; *p* < 0.0012) than the LQP and LP offspring, respectively (Figure 3G). Body length at the end of the experiment was significantly lower in the LQP (*p* < 0.0001) and LP (*p* < 0.0001) than the NP offspring (Figure 3H). However, body length was significantly higher in the LQPR (*p* < 0.0001) and LPR (*p* < 0.004) than LQP and LP offspring at the end of the experiment (Figure 3H).

#### 3.2.3. Whole-Body Composition of the Offspring

The whole-body composition parameters at weaning (Table 3) and 10 weeks post-weaning (Table 4) were recorded using DEXA. The total body surface area, BMC, total mass, lean mass, fat mass, and % BF were significantly lower in the LQP and LP than in the NP offspring at weaning. The percent lean body mass was significantly higher in the LQP (*p* < 0.0001) and LP (*p* < 0.0004) than the NP offspring. LQP and LP offspring had a significantly lower total body surface area, BMC, BMD, total mass, lean mass, and fat mass than the NP offspring at 10 weeks after weaning. Percent LBM was significantly higher in the LQP (*p* < 0.0001) and remained unchanged in LP compared to the NP offspring. Percent BF was significantly lower in the LQP (*p* < 0.0001) and remained unchanged in LP compared to the NP offspring. The total body surface area, BMC, BMD, total mass, and lean mass were significantly higher in the LQPR and LPR than the LQP and LP offspring, respectively. Furthermore, %LBM, fat mass and %BF were significantly higher in the LQPR (*p* < 0.05) than the LQP offspring. Although fat mass was higher in the LPR than in the LP offspring, the results remained insignificant.

#### 3.2.4. Absolute and Relative Organ Weights of the Offspring

The absolute and relative (proportional to BWs) weights of the vital organs are shown in Table 5. The absolute weights of the vital organs in the LQP and LP—including the brain (20% and 15%), liver (72% and 39%), kidney (69% and 55%), heart (57% and 30%), and pancreas (63% and 40%)—were significantly lower than those in the NP offspring. Conversely, the relative weights of the brain (217%), kidney (27%), heart (73%), and pancreas (55%) were significantly higher in the LQP than the NP offspring. Furthermore, the relative weights of the brain (71%), liver (27%), and heart (34%) were significantly higher in the LP than in the NP offspring. However, the absolute weights of the brain (14%), liver (46%), kidney (41%), heart (39%), and pancreas (45%) in the LQPR, and the liver (27%), kidney (28%), and pancreas (22%) in the LPR were significantly higher than those in the LQP and LP offspring, respectively. Furthermore, the relative weights of the brain (180%) and heart (55%) in LQPR, and the brain (71%), liver (27%), and heart (34%) in the LPR were significantly higher than those in the LQP and LP offspring, respectively.

#### 3.2.5. Lipid Profile

**Figure 4:**
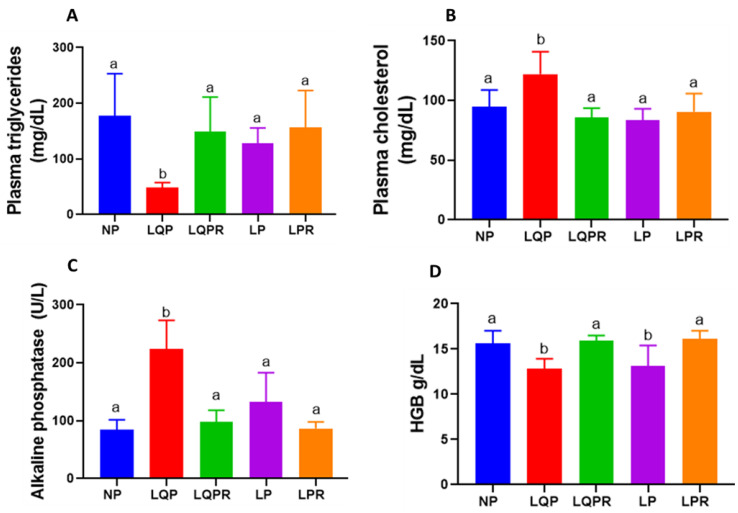
Effect of maternal protein restriction followed by rehabilitation on the lipid profile, alkaline phosphatase (ALP), and hemoglobin (Hb) in the offspring: the lipid—including triacylglycerides (TAG), cholesterol, and ALP in the plasma and Hb in the total blood of the offspring—was estimated at the end of the experiment. (**A**) TAG levels, (*n* = 8 per group), (**B**) cholesterol levels (*n* = 8 per group), (**C**) ALP levels (*n* = 8 per group), and (**D**) Hb levels (*n* = 8 per group, except NP; *n* = 7). The bars indicate the mean ± SD, and the bars that have different superscript letters differ significantly (*p* < 0.05). NP, normal protein; LQP, low-quality protein; LQPR; low-quality protein group rehabilitated with the NP diet; LP, low protein; LPR, low-protein group rehabilitated with the NP diet.

The plasma TAG levels were significantly lower (*p* < 0.0003) and the cholesterol levels were higher (*p* < 0.002) in the LQP than the NP offspring (Figure 4A,B). The triacylglycerides and cholesterol levels remained unaltered in the LP compared to the NP offspring. However, the TAG levels were significantly higher (*p* < 0.007) and the cholesterol levels were lower (*p* < 0.0001) in the LQPR compared to the LQP offspring (Figure 4A,B).

#### 3.2.6. Alkaline Phosphatase and Hemoglobin

The alkaline phosphatase levels were significantly higher in the LQP (*p* < 0.0001) and the LP (*p* < 0.05) than the NP offspring (Figure 4C). However, the ALP levels were significantly lower in LQPR (*p* < 0.0001) and unaltered in LPR (*p* < 0.07) compared to the LQP and LP offspring, respectively (Figure 4C). The hemoglobin levels were significantly lower in the LQP (*p* < 0.003) and LP (*p* < 0.01) than the NP offspring (Figure 4D). However, the Hb levels were significantly higher in the LQPR (*p* < 0.0007) and LPR (*p* < 0.001) compared to the LQP and LP offspring, respectively (Figure 4D).

#### 3.2.7. Glucose Homeostasis and HOMA-IR

**Figure 5:**
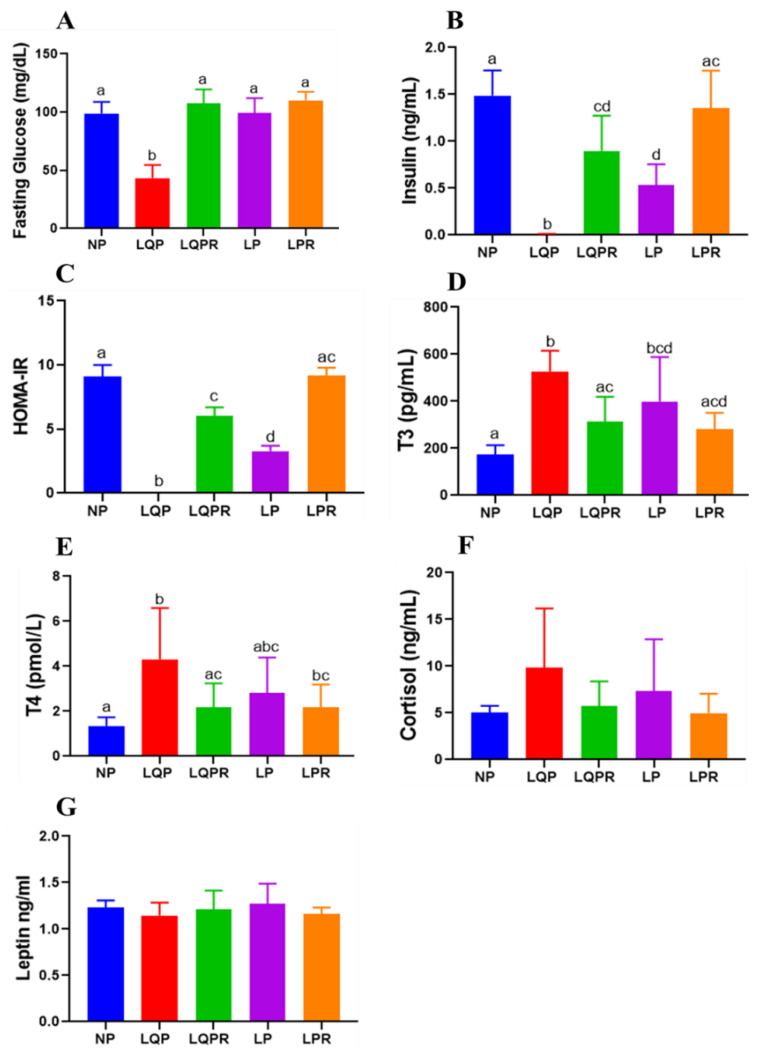
Effect of maternal protein restriction followed by rehabilitation on the glucose homeostasis and hormone profile in the offspring: fasting glucose, insulin, T3, T4, and cortisol were estimated in the offspring’s plasma at the end of the experiment. (**A**) Fasting glucose levels, (*n* = 8 per group), (**B**) insulin levels, (*n* = 6 per group), (**C**) HOMA-IR (*n* = 6 per group), (**D**) T3 levels (*n* = 6 per group), (**E**) T4 levels (*n* = 6 per group), (**F**) cortisol levels (*n* = 6 per group), and (**G**) leptin levels (*n* = 8 per group). The bars indicate the mean ± SD, and the bars that share different superscript letters differ significantly (*p* < 0.05). NP, normal protein; LQP, low-quality protein; LQPR; low-quality protein group rehabilitated with the NP diet; LP, low protein; LPR, low-protein group rehabilitated with the NP diet.

The plasma FG levels were significantly lower in the LQP (*p* < 0.0001) and remained unaltered in the LP compared to the NP offspring (98.80 ± 10 mg/dL; Figure 5A). However, the FG levels were significantly higher in the LQPR (*p* < 0.0001) than the LQP offspring (Figure 5A). The insulin levels were remarkably lower in the LQP (*p* < 0.0001) and LP (*p* < 0.0001) than the NP offspring (1.48 ± 0.26 ng/mL, Figure 5B). Nevertheless, the insulin levels were significantly higher in the LQPR (*p* < 0.0002) and LPR (*p* < 0.0003) than the LQP and LP offspring (Figure 5B). HOMA-IR was not determined in the LQP (*p* < 0.0001) due to the much lower glucose and insulin levels, while it was significantly lower in the LP (*p* < 0.01) than the NP offspring (Figure 5C). However, HOMA-IR was significantly higher in the LQPR (*p* < 0.01) and LPR (*p* < 0.01) than the LQP and LP offspring, respectively (Figure 5C).

#### 3.2.8. Hormone Profile

The plasma T3 levels were significantly higher in the LQP (*p* < 0.0001) and LP (*p* < 0.0043) than the NP offspring (Figure 5D). However, the realimented NP diet significantly increased the T3 levels in the LQPR (*p* < 0.012), and tended to be increased in LPR compared to the LQP and LP offspring. In the same manner, the plasma T4 levels were also significantly higher in the LQP (*p* < 0.0001) and remain unchanged in the LP compared to the NP offspring (Figure 5E). The realimented NP diet significantly increased the T4 levels in the LQPR (*p* < 0.0003) compared to the LQP offspring. There was no significant difference in the cortisol and leptin levels between the offspring groups (Figure 5F,G).

## 4. Discussion

### 4.1. Imapct of the MLQP and MLP Diets in the Dams

In the present study, we shed light on the effect of long-term quality and quantity protein restriction, starting seven weeks before conception and continuing until one-month post-lactation in the mothers. We continued the mothers until one-month after lactation to determine the long-term effects in the mothers in addition to the offspring. LQP and LP diets promoted a slight BW gain without altering the food intake. Accumulating evidence suggests that LP diets are associated with increased BW, hyperphagia, energy expenditure and thermogenesis [37]; however, the increase in BWs was slighter, and may have been due to an increase in fat mass. The increase in fat mass in our study was possibly attributable to the compensation for the loss of lean tissue. The lower % LBM in dams exposed to LP diets can be explained by dietary protein dilution and the increased mobilization of proteins from the muscle in response to increased demands for the development of pups during gestation, and milk production during lactation [38]. The lower availability of IAA in dams exposed to LQP diets may explain the lower lean mass. The plasma TAG levels were marginally increased substantiating with the increased fat mass.

### 4.2. Imapct of MLQP and MLP Diets in the Offspring

The long-term effects of maternal quality and quantity protein restriction were investigated in the offspring, which were maintained until four months after lactation. The important findings of the study are as follows: chronic MLQP and MLP diets (i) profoundly reduced the BWs by lowering LBM and fat mass, and by promoting hypermetabolism; and (ii) enhanced insulin sensitivity by promoting hypoglycemia and hypoinsulinemia in the offspring. (iii) The rehabilitation of LQP and LP offspring with the NP diet from weaning induced catch-up growth by completely or partially reversing the above outcomes. (iv) The thyroid profile, lipid profile, %LBM, %BF, FG, and relative weight of a few organs showed the differential effect in MLQP and MLP diets. Overall, we report that MLP and MLQP diets during combined periods of preconception to post-weaning possibly induce differential adverse effects, and that these effects are reversed with the feeding of an NP diet from weaning.

To our knowledge, this is the first comprehensive study assessing the effect of chronic MLQP and MLP diets on the body composition, thyroid profile, Hb, ALP, and differential effects on body phenotype. Earlier studies reported the impact of the MLQP diet using WG on the amino acid levels in the blood, brain [39], and mammary glands [39] in mothers, and BW [32] in the offspring. In contrast to the dams, the LQP offspring had lower BW, LBM, and fat mass, and exhibited decreases in BMD and BMC, demonstrating severe growth retardation. The gain in BW and fat mass in the mothers along with the reduction in offspring imply that quality PR during early and adult life may have distinct metabolic phenotypes.

Previous studies reported that exposure to an MLQP diet containing WG during gestation and lactation caused no change in BW in the offspring [32]. In our study, chronic restriction of quality protein from preconception (7 weeks) to 16 weeks after weaning showed a remarkably lower BW, demonstrating that the effects of maternal undernutrition are dependent on timing and the length of PR. However, BW was significantly improved in rehabilitated groups (LQPR and LPR), indicating the stimulation of catch-up growth. Similarly, the reduction of BW in the offspring of mothers born on an LP diet, from preconception to weaning, was also higher than earlier studies in which LP diets were fed during gestation and lactation only [40]. Interestingly, LQP and LP offspring had lower BW despite their higher relative food intake across the first few weeks.

Further, BW was lowered even though the relative food intake was comparable between the groups at the end of the experiment. Decreased satiety in the LQP and LP offspring in the initial stages, and adaptation in the later stages, may explain the altered relative food intake. The BW of the offspring born to mothers fed on a turkey and pea protein diet in gestation through the lactation and post-weaning was also lower than the offspring of mothers fed on a milk protein diet [34]. In another study, BW was higher in the offspring of mothers who were fed a soy protein diet during gestation and lactation [17]. It has been reported that AAs—including alanine, leucine, isoleucine, methionine, arginine, valine, and lysine—are lower in WG compared to casein [33]. Earlier, it was shown that the deprivation of essential AAs, including lysine and methionine, affect blastocyst development by altering embryonic stem cell proliferation [41,42].

Moreover, the deprivation of lysine and methionine in the diet impairs the offspring’s growth [43,44]. Rehabilitation ameliorated the BW by averting the 44% and 29% of reduction in the LQPR and LPR compared to LQP and LP offspring. Therefore, the supply of maternal diets inadequate in essential AAs in the form of the quality and quantity of a protein affects the fetal development and subsequent health of the offspring.

MLQP and MLP intake showed decreased BMC and BMD in the offspring. The decreased BMC and BMD is one of the causative factors for lower BWs and body length. Protein restriction is associated with a reduction in intestinal calcium absorption and IGF-1 secretion, and an increase in parathyroid hormones [45]. The long-term intake of MLQP and MLP diets may induce changes in bone metabolism in the offspring. In support of this, several epidemiological studies reported reduced BMD and increased bone loss in individuals consuming LP diets [46].

The proportional increase of organ weights relative to BWs in the LQP and LP offspring suggests that animals are physiologically adapted to maintain enhanced functional capacity in harsh environmental conditions at the expense of growth, and to maximise the chances of survival. It is noteworthy that the relative weight of the vital organs, including the brain, was remarkably higher (217 and 73%), followed by heart (73 and 43%) in LQP and LP offspring, supporting the brain-sparing hypothesis in which blood flow towards the brain is preferentially increased to preserve the brain development during placental insufficiency [47,48]. The relative weights of the pancreas, kidney, and liver varied differentially between the LQP and LP offspring. The higher relative weight of the pancreas (55%) and kidney (27%) in LQP without any change in the LP offspring highlights that the impact of inadequate quality protein is more severe than the LP. Adding to this, the higher relative liver weight in LP (27%) without any change in the LQP offspring may suggest that the LP diet may increase the risk of developing a fatty liver, rather than poor protein sources. Previous studies have also reported that the MLP diet induces a fatty liver in offspring [49]. These observations further highlight the selective organ growth in chronic quality and quantity protein restriction. Previous studies have shown selective organ growth in offspring born to mothers exposed to an LP diet [48], but not in mothers exposed to an LQP diet.

Hypoglycemia in LQP, hypoinsulinemia and lower HOMA-IR in LQP and LP offspring suggests that these animals had enhanced insulin sensitivity. Maternal LQP and LP diets enhance insulin sensitivity at the early stages of growth in the offspring, and may pave the way for insulin resistance in later stages [50]. Rehabilitation completely restored the FG levels in LQPR compared to those of the NP offspring. The insulin levels were still lower in the LQPR offspring despite the complete restoration of the FG levels and weight of the pancreas.

In contrast to earlier studies, the ALP levels were elevated in the LQP and LP offspring. Earlier studies reported differential results with decreased levels of ALP in rat plasma [51] and mouse osteoblasts [52], along with increased levels in monkey offspring born to LP mothers [53]. Decreased Hb levels in LP offspring suggest that exposure to an MLP diet for a long period (pre-pregnancy to post-weaning) may pose a risk for the development of anaemia. In support of this, an earlier study reported that the MLP diet alters the hemopoietic microenvironment and globin synthesis [54].

Hypermetabolism due to higher levels of thyroid hormones in LP offspring may be a contributing factor for lower BWs in the offspring. Our findings are further corroborated by previous observations showing that an MLP diet during gestation and/or lactation affects thyroid function and BWs [55,56]. The mild increase in cortisol levels in the LQP and LP offspring possibly suggests the offspring’s susceptibility to physiological and psychological stress. It should be noted that the food intake was altered without any change in leptin levels. Several past studies have reported no change in leptin levels in offspring exposed to prenatal and postnatal protein restriction [33,53]. Therefore, there is a need to estimate orexigenic hormones, which will provide additional information regarding alterations in food intake.

The recovery of several factors upon rehabilitation with the NP diet from weaning showed varied grades, ranging from partial to complete restoration. Metabolic factors including FG, T3, T4, TAG, cholesterol, ALP, HB, body length, and organ weights are restored either nearer or completely to normal levels, while BWs, body composition, insulin levels, and HOMA-IR are partially restored to normal levels upon rehabilitation in LQPR offspring. Rehabilitation in the LPR offspring showed the restoration of the insulin, HOMA-IR, T3, Hb, and organ weights—except for body length—either nearer or completely to normal levels, while BWs and body composition were partially restored to normal levels. These observations highlight the reversible changes upon rehabilitation of offspring born to protein-restricted (quality and content) mothers with the NP diet from weaning.

Previous studies have debated whether rapid weight gain during catch-up growth may contribute to adiposity and the risk of metabolic diseases in later life [57,58]. However, our study is limited by duration, as we followed it up to 4 months after weaning. A few past studies have shown that the development of insulin resistance in offspring is present in 15 months and above aged offspring born to LP-mothers [59]. Furthermore, our study is limited by sex-dependent effects for the terminal parameters.

Our study led to two important observations that the outcomes of maternal quality and quantity PR during combined periods of preconception to post-weaning were more profoundly affected than the PR during gestation and/or lactation only. The effects of maternal quality protein restriction were more severe than the maternal dietary protein dilution in terms of outcome. The outcomes of our study should be analyzed by considering the difference in the percentage of the macromolecule in the isocaloric LP and LQP diets, as dietary proteins are diluted with a high carbohydrate content in LP diets, while LQP diets contain a normal carbohydrate content. Therefore, the outcomes of MLP diets are attributed to the LP-high carbohydrate content and to the LQP content alone in MLQP diets.

## 5. Conclusions

In conclusion, we report that chronic maternal quality protein restriction and dietary protein dilution induce differential adverse effects by programming the body composition and metabolism, followed by reversible changes upon rehabilitation with NP, which may have implications in infant and young child supplementary nutrition programs in developing countries. Our study will likely provide metabolic and biochemical insights into the long-term effects of maternal dietary protein dilution and the maternal restriction of quality protein in the offspring. Furthermore, our study paves the way for the investigation of the impact of various quality proteins and IAA during gestation and lactation in the offspring.

## Figures and Tables

**Table 1:** Composition of the diets used in the study.

Composition	NP	LQP	LP
Metabolizable energy (kcal/g of diet)	3.9	3.9	3.9
kJ/g of diet	16.3	16.3	16.3
Proteins (% energy)	20	20	8
Carbohydrates (% energy)	64	64	76
Lipids (% energy)	16	16	16
Ingredients (g/kg of diet)			
Starch	495	495	536.5
Casein	200	0	80
Wheat gluten	0	200	0
Sucrose	132.5	132.5	212.5
Ground Nut Oil	70	70	70
Cellulose	50	50	50
Mineral Mix (AIN-93G-MX) ^1^	35	35	35
Vitamin Mix (AIN-93-VX) ^2^	10	10	10
L.Cystine	3	3	1.5
Choline Chloride	2.5	2.5	2.5
PABA	1	1	1
Inositol	1	1	1

^1^ Mineral mix (AIN-93G-MX): MP Biomedicals, LLC (C.No.960400). The mineral mix contained (%): CaCO3, 35.7%; KH2PO4, 19.6%; C6H7K3O8, 7.078%; NaCl, 7.4%; K2SO4, 4.66%; MgO, 2.4%; C6H5FeO7, 0.606%; ZnCO3, 0.165%; MnCO3, 0.063%; CuCO3, 0.03%; KIO3, 0.001%; Na2SeO4, 0.00103%; (NH4)6Mo7O24.4H2O, 0.000795%; Na2SiO3.9H2O, 0.145%; KCr(SO4)2.12H2O, 0.0275%; LiCl, 0.00174%; H3BO3, 0.008145%; NaF, 0.00635%: NiCO3, 0.00318%; NH4VO3, 0.00066%; and powdered sugar, 22.1%. ^2^ Vitamin mix (AIN-93-VX): MP Biomedicals, LLC (C.No.960402). The vitamin mixture contained (g/kg): Nicotinic Acid, 3.00 g; D-Calcium Pantothenate, 1.60 g; Pyridoxine HCl, 0.70 g; Thiamine HCl, 0.60 g; Riboflavin, 0.60 g; Folic Acid, 0.20 g; D-Biotin, 0.02 g; Vitamin B12 (0.1% triturated in mannitol), 2.50 g; a-Tocopherol Powder (250 U/gm), 30.00 g; Vitamin A Palmitate (250,000 U/gm) 1.60 g; Vitamin D3 (400,000 U/gm), 0.25 g; Phylloquinone, 0.075 g; and powdered Sucrose, 959.65 g. NP, normal protein; LQP, low-quality protein; LP, low-protein diet.

**Table 2:** Effect of quality and quantity protein restriction on the whole-body composition and metabolic factors in mothers.

Four Weeks	NP (*n* = 4)	LQP (*n* = 4)	LP (*n* = 4)
Area (cm^2^)	45.68 ± 3.65	47.06 ± 1.20	46.64 ± 1.20
BMC(g)	7.24 ± 0.44	7.39 ± 0.43	7.28 ± 0.25
BMD (g/cm^2^)	0.15 ± 0.004	0.15 ± 0.003	0.15 ± 0.004
Total mass (g)	197.72 ± 16.17	211 ± 13.65	211.77 ± 12.97
Lean mass (g)	148.80 ± 8.87	150.58 ± 2.46	147.88 ± 4.12
% LBM	79.07 ± 3.42	75.06 ± 4.17	73.41 ± 3.40
Fat mass (g)	41.7 ± 9.63	53.05 ± 11.77	56.57 ± 10.34
% BF	20.95 ± 3.41	24.95 ± 4.18	26.5 ± 3.40
Fasting Glucose (mg/dL)	77.25 ± 6.07	74.5 ± 5.97	73.75 ± 7.5
At weaning			
Area (cm^2^)	48.02 ± 2.30	48.92 ± 1.88	49.4 ± 2.07
BMC(g)	6.95 ± 0.54	7.24 ± 0.56	7.21 ± 0.51
BMD (g/cm^2^)	0.14 ± 0.005	0.148 ± 0.007	0.146 ± 0.007
Total mass (g)	207.15 ± 25.34	207.27 ± 17.01	229.02 ± 13.61
Lean mass (g)	180.32 ± 18.94	160.82 ± 8.43	173.6 ± 3.05
% LBM	90.58 ± 2.17 ^a^	81.22 ± 2.35 ^b^	79.12 ± 4.08 ^b^
Fat mass (g)	19.85 ± 6.3 ^a^	39.22 ± 8.35 ^b^	48.2 ± 11.74 ^b^
% BF	9.4 ± 2.18 ^a^	18.77 ± 2.33 ^b^	20.87 ± 4.11 ^b^
Fasting Glucose (mg/dL)	80.5 ± 12.12	62.75 ± 3.30	72.5 ± 12.92
Plasma cholesterol (mg/dL)	88.61 ± 15.84	84.81 ± 22.87	76.23 ± 8.69
Plasma triglycerides (mg/dL)	85.43 ± 55.01	126.37 ± 66.19	230.31 ± 95.09

The whole-body composition of the mothers was assessed by dual-energy X-ray absorptiometry scanning at weaning. The data represent the mean ± SD, (*n* = 4 per group). Mean values that share different superscript letters differ significantly from each other (*p* < 0.05). NP, normal protein; LQP, low-quality protein; LP, low-protein diet.

**Table 3:** Effect of maternal quality and quantity protein restriction on whole-body composition and metabolic factors in the offspring at weaning.

	NP (*n* = 6)	LQP (*n* = 6)	LP (*n* = 6)
Area (cm^2^)	26.2 ± 1.82 ^a^	4.32 ± 0.59 ^b^	7.84 ± 1.20 ^c^
BMC(g)	2.15 ± 0.14 ^a^	0.34 ± 0.04 ^b^	0.66 ± 0.10 ^c^
BMD (g/cm^2^)	0.082 ± 0.001 ^ab^	0.080 ± 0.003 ^a^	0.085 ± 0.02 ^b^
Total mass (g)	98.4 ± 5.94 ^a^	12.18 ± 1.53 ^b^	25.43 ± 2.53 ^c^
Lean mass (g)	78.17 ± 5.07 ^a^	10.93 ± 1.17 ^b^	21.80 ± 2.00 ^c^
% LBM	81.64 ± 2.06 ^a^	92.79 ± 2.64 ^b^	88.40 ± 2.13 ^c^
Fat mass (g)	18.08 ± 2.28 ^a^	0.91 ± 0.40 ^b^	2.95 ± 0.65 ^b^
% BF	18.4 ± 2.05 ^a^	7.28 ± 2.56 ^b^	11.58 ± 2.15 ^b^

The whole-body composition of the offspring was assessed by dual-energy X-ray absorptiometry scanning at weaning. The data represent the mean ± SD (*n* = 6 per group). Mean values that share different superscript letters differ significantly from each other (*p* < 0.05). NP, normal protein; LQP, low-quality protein; LP, low-protein.

**Table 4:** Effect of maternal quality and quantity protein restriction followed by rehabilitation on whole-body composition and metabolic factors in the offspring at 10 weeks after weaning.

	NP (*n* = 6)	LQP (*n* = 6)	LQPR (*n* = 6)	LP (*n* = 6)	LPR (*n* = 6)
Area (cm^2^)	57.48 ± 8.33 ^a^	15.02 ± 3.39 ^b^	44.29 ± 5.12 ^c^	30.81 ± 2.57 ^d^	46.92 ± 8.28 ^c^
BMC(g)	8.43 ± 1.32 ^a^	1.51 ± 0.38 ^b^	6.05 ± 0.92 ^c^	3.33 ± 0.42 ^d^	6.5 ± 1.16 ^c^
BMD (g/cm^2^)	0.14 ± 0.007 ^a^	0.10 ± 0.01 ^b^	0.13 ± 0.006 ^a^	0.10 ± 0.006 ^b^	0.13 ± 0.007 ^a^
Total mass (g)	311.58 ± 64.93 ^a^	52.2 ± 9.38 ^b^	205.75 ± 49.25 ^c^	119.45 ± 18.03 ^b^	234.11 ± 55.01 ^cd^
Lean mass (g)	233.74 ± 49.05 ^a^	45.48 ± 8.06 ^b^	164.06 ± 39.9 ^c^	87.48 ± 12.2 ^b^	180.71 ± 43.53 ^ac^
% LBM	77.76 ± 2.77 ^a^	90.13 ± 4.49 ^b^	82.83 ± 4.07 ^ac^	76.19 ± 2.21 ^ac^	79.92 ± 1.85 ^ac^
Fat mass (g)	69.41 ± 18.36 ^a^	5.21 ± 2.85 ^b^	35.63 ± 12.52 ^c^	28.65 ± 5.98 ^c^	46.88 ± 11.37 ^c^
% BF	22.23 ± 2.75 ^a^	9.91 ± 4.42 ^b^	17.16 ± 4.05 ^ac^	23.81 ± 2.1 ^ac^	20.08 ± 1.87 ^ac^

The whole-body composition of the offspring was assessed by dual-energy X-ray absorptiometry scanning at 10 weeks after weaning. The data represent the mean ± SD. (*n* = 6 per group). Mean values that share different superscript letters differ significantly from each other (*p* < 0.05). NP, normal protein; LQP, low-quality protein; LQPR, low-quality protein group rehabilitated with the NP diet; LP, low-protein; LPR, low-protein group rehabilitated with the NP diet.

**Table 5:** Effect of maternal quality and quantity protein restriction followed by rehabilitation on the organ weights in the offspring.

Organs	NP (*n* = 8)	LQP (*n* = 8)	LQPR (*n* = 8)	LP (*n* = 8)	LPR (*n* = 8)
Bodyweight (BW; g)	353.12 ± 78.09 ^a^	85.62 ± 15.37 ^b^	241. ± 63.43 ^c^	168.12 ± 22.75 ^c,d^	272.5 ± 57.07 ^c^
Brain (g)	1.8 ± 0.12 ^a^	1.43 ± 0.13 ^b^	1.69 ± 0.16 ^a,c^	1.52 ± 0.13 ^b,c,d^	1.69 ± 0.13 ^a,c,d^
Liver (g)	12.05 ± 2.87 ^a^	3.35 ± 0.06 ^b^	8.88 ± 2.24 ^c^	7.2 ± 1.04 ^c,d^	10.53 ± 2.5b ^a,c^
Kidney (g)	2.62 ± 0.6 ^a^	0.8 ± 0.15 ^b^	1.88 ± 0.47 ^c^	1.17 ± 0.14 ^b^	1.89 ± 0.34 ^c^
Heart (g)	1.12 ± 0.15 ^a^	0.47 ± 0.07 ^b^	0.91 ± 0.19 ^a,c^	0.79 ± 0.15 ^c^	0.95 ± 0.14 ^a,c^
Pancreas (g)	0.81 ± 0.3 ^a^	0.29 ± 0.08 ^b^	0.66 ± 0.2 ^a,c^	0.43 ± 0.12 ^b,c,d^	0.61 ± 0.19 ^a,c,d^
Brain weight/BW (%)	0.53 ± 0.13 ^a^	1.7 ± 0.19 ^b^	0.73 ± 0.16 ^a,c^	0.91 ± 0.14 ^cd^	0.64 ± 0.15b ^a,c^
Liver weight/BW (%)	3.41 ± 0.24 ^a^	3.9 ± 0.61 ^ab^	3.7 ± 0.22 ^a^	4.36 ± 0.66 ^b^	3.83 ± 0.32 ^a,b^
Kidney weight/BW (%)	0.74 ± 0.06 ^a^	0.94 ± 0.11 ^b^	0.78 ± 0.03 ^a^	0.7 ± 0.07 ^a^	0.7 ± 0.04 ^a^
Heart weight/BW (%)	0.32 ± 0.04 ^a^	0.56 ± 0.08 ^b^	0.38 ± 0.04 ^a^	0.46 ± 0.05 ^c^	0.35 ± 0.03 ^a^
Pancreas weight/BW (%)	0.22 ± 0.06 ^a^	0.35 ± 0.11 ^b^	0.28 ± 0.1 ^a,b^	0.25 ± 0.07 ^a,b^	0.22 ± 0.04 ^a^

The organ weights of the offspring were recorded during the sacrifice. The data represent the mean ± SD, (*n* = 8 per group). Mean values that share different superscript letters differ significantly from each other (*p* < 0.05). NP, normal protein; LQP, low-quality protein; LQPR, low-quality protein group rehabilitated with the NP diet for recovery; LP, low protein; LPR, low-protein group rehabilitated with the NP diet.

## Data Availability

The data presented in this study are available with corresponding author upon request.
